# Assessing the Need for Mobile Health (mHealth) in Monitoring the Diabetic Lower Extremity

**DOI:** 10.2196/11879

**Published:** 2019-04-16

**Authors:** David Wallace, Julie Perry, Janelle Yu, Joshua Mehta, Paul Hunter, Karen Michelle Cross

**Affiliations:** 1 Division of Plastic and Reconstructive Surgery University of Toronto Toronto, ON Canada; 2 Faculty of Medicine University of Toronto Toronto, ON Canada; 3 Division of Plastic Surgery St. Michael's Hospital Toronto, ON Canada; 4 Keenan Research Centre for Biomedical Science Toronto, ON Canada

**Keywords:** mHealth, diabetes, diabetic foot ulcers

## Abstract

**Background:**

Complications of the diabetic lower extremity (such as diabetic foot ulcers, DFUs) occur when monitoring is infrequent, and often result in serious sequelae like amputation or even death.

**Objective:**

To evaluate the potential application of mobile health (mHealth) to diabetic foot monitoring. We surveyed the self-management routines of a group of diabetic patients, as well as patient and clinician opinions on the use of mHealth in this context.

**Methods:**

Patients with DFUs in Toronto, Ontario, Canada completed a 25-item questionnaire addressing their foot care practices, mobile phone use, and views on mHealth. Wound care clinicians across Canada were also surveyed using a 9-item questionnaire.

**Results:**

Of the patients surveyed, 59/115 (51.3%) spend less than a minute checking their feet, and 17/115 (15%) of patients find it difficult to see their doctor or get to the hospital regularly. Mobile phone use was widespread in our patient cohort (93/115, 80.9%). Of mobile phone users, 68/93 (73.1%) would use a device on their mobile phone to help them check their feet. Of the clinicians who completed the questionnaire, only 7/202 (3.5%) were familiar with mHealth; however, 181/202 (92%) of clinicians expressed interest in using mHealth to monitor their patients between visits.

**Conclusions:**

Patient education or motivation and clinician training were identified as the major barriers to mHealth use in the diabetic lower extremity, which may be a viable mechanism to improve DFU monitoring practices.

## Introduction

The burden of treatment in diabetes is high: patients must monitor their diet and blood glucose levels, take medication, refill prescriptions, travel to medical appointments, seek information, and keep records [1]. This “illness work” can become time- and identity-consuming, and can impose so much on a patient’s everyday life that treatment compliance rates drop and outcomes decline [1]. One common complication that can result from patient burn-out are diabetic foot ulcers (DFUs) due to a combination of vascular problems of the lower extremity and the lack of sensation that is common in diabetes (diabetic neuropathy). An ill-fitting sock or shoe can rub enough to cause a foot ulcer in a diabetic person, which may go unnoticed until it is large or infected. When caught early, DFUs are highly treatable; however, most ulcers are not treated until they become more advanced, such that one third of ulcers never heal and result in foot or lower extremity amputation [2,3]. The 3-year overall mortality rate following lower extremity amputation due to DFUs is as high as 70% [4]—higher than cancers of the breast and colon. These devastating outcomes are preventable, but consistent monitoring of foot health and early reporting of problems is critical.

The concept of *minimally disruptive medicine* (MDM) recognizes the workload associated with a chronic illness, and aims to simplify, consolidate, and synchronize healthcare activities to help patients manage their conditions efficiently [5]. A growing area of research with application to MDM is the delivery of healthcare remotely via apps on mobile devices (called mobile health or mHealth). Mobile phones are ubiquitous in society, and several software platforms have been developed for the self-management of diabetes. Much of mHealth’s application to diabetes has been focused on apps that track patient blood glucose levels at home. Fewer hyperglycemic events and lower average glucose levels were reported in patients using mobile health platforms than non-mobile health-using controls [6]. There is currently no mHealth tool for diabetic foot health monitoring, although there is evidence that monitoring via telemedicine is as effective as standard outpatient monitoring in regards to healing and amputation rate [7]. Of note, however, Rasmussen et al observed a significantly higher mortality rate in the telemedicine group in their study, with no easily ascribable cause. Therefore, while highly promising, adoption of mHealth monitoring practices should proceed with caution.

To determine if an MDM-based mHealth intervention is needed for the diabetic lower extremity, we sought to characterize the daily self-management routines of diabetics at St. Michael’s Hospital (SMH) in Toronto, Ontario, Canada, and the attitudes towards mHealth among Canadian foot care clinicians. Our goal was to understand the barriers to implementing such a strategy from both sides of the patient-clinician continuum.

## Methods

### Patient Questionnaire

This was a descriptive study of patients with DFUs presenting to plastic surgery clinics at SMH in Toronto over a 6-month period in 2017. SMH is a large, tertiary, academic level-1 trauma centre located in the downtown core. This study was approved by the SMH Research Ethics Board (REB 17-023). The questionnaire was designed by clinicians who treat this patient population, but was not validated in any way.

Patients at SMH were approached by study investigators following clinic visits and informed about the study. Patients were given the opportunity to ask questions, and if they chose to participate, they signed informed consent.

The study investigators designed a questionnaire addressing multiple themes associated with patients with DFUs, including characteristics of patient health, mobile phone use, and views on mHealth (see Multimedia Appendix 1). The majority of questions were designed to have “yes” or “no” answers or asked participants to “rate your experience from 1 to 10” in order to facilitate a shorter clinic experience for the patient. Patients were not given the option to add comments. The questionnaire was comprised of 25 questions and was designed to take patients approximately 5 minutes to complete. All responses from the patients surveyed were inputted into an electronic database and coded anonymously using a unique patient identifier. All continuous data were reported using means, medians, modes, and ranges. All categorical data were reported using frequencies and proportions. All analysis was carried out using Microsoft Excel.

### Healthcare Provider Questionnaire

A 9-question survey (see Multimedia Appendix 1) was designed in Survey Monkey and distributed via email to the membership of Wounds Canada following Research Ethics Board approval at SMH. Responses were collected for 4 weeks, after which the survey was terminated. No personal identifiers were collected to link respondents to their responses.

## Results

### Patient Survey

Of the 117 patients who were approached in plastic surgery and diabetes clinics at SMH, 115 agreed to be asked a series of qualitative questions describing their foot checking practices and comfort with mobile technology. The average age of participants was 54.8 years, and 60/115 (52.2%) of the participants were men (Table 1). Of the participants, 68/115 (59.1%) were Type 2 diabetics, 91/115 (79.1%) were insulin dependent, and 108/115 (93.9%) used a glucometer. The average BMI of patients was 28.2 kg/m^2^. Of the participants, 100/115 (87.0%) were non-smokers, 91/115 (79.1%) stated that they visit their physician a few times a year, and most report that being in control of their own health is very important to them (mean rating of 8.3 out of 10, where 10 is the highest importance; Table 1).

When asked about their current foot checking practices, 89/115 (77.4%) of patients reported checking their feet regularly, although 59/115 (51.3%) reported spending less than a minute checking, and only 16/115 (13.9%) use a mirror to check the bottoms of their feet (Table 1). Most patients (103/115, 89.6%) reported being comfortable touching their toes (suggesting that they are flexible enough to bend and check their feet), but 83/115 (72.2%) of respondents reported wearing corrective lenses, which may affect their ability to see their feet clearly. Importantly, only 11/115 (9.6%) of participants reported a previous DFU, and only 3/115 (2.6%) of patients had a prior amputation involving their toe or leg (Table 1).

**Table 1:** Summary of patient demographic data and survey (n=115).

Patient demographic or survey response		Responses
Age, mean (range)		54.8 (18-84)
**Gender, n (%)**		
	Male	60 (52.2)
	Female	60 (52.2)
	Unknown	5 (4.4)
Body mass index (kg/m^2^), mean (SD)		28.17 (7.91)
**Occupation, n (%)**		
	Administration or management	8 (7.0)
	Education	7 (6.1)
	Engineering or technology	6 (5.2)
	Finance or business	21 (18.3)
	Healthcare	4 (3.5)
	Labor	4 (3.5)
	Other	17 (14.8)
	Retired	30 (26.1)
	Student	3 (2.6)
	Unemployed	13 (11.3)
	No answer	2 (1.7)
**Diabetes type, n (%)**		
	1	42 (36.5)
	2	68 (59.1)
	Not sure	6 (5.2)
**Uses insulin, n (%)**		
	Yes	91 (79.1)
	No	23 (20.0)
	No answer	1 (0.8)
**Smoking status, n (%)**		
	Smoker	12 (10.4)
	Never smoker	100 (87.0)
	No answer	3 (2.6)
**Wears corrective lenses, n (%)**		
	Yes	83 (72.2)
	No	32 (27.8)
**Comfortable touching their toes, n (%)**		
	Yes	103 (89.6)
	No	8 (7.0)
	No answer	4 (3.5)
**Has had a prior diabetic foot ulcer, n (%)**		
	Yes	11 (9.6)
	No	101 (87.8)
	No answer	3 (2.6)
**Has had a toe or leg amputation, n (%)**		
	Yes	3 (2.6)
	No	112 (97.4)
	No answer	0 (0)
Importance of being in control of their own health^a^, mean (SD)		8.28 (2.37)
**Has difficulty getting to the hospital or seeing their doctor, n (%)**		
	Yes	17 (14.8)
	No	95 (82.6)
	No answer	3 (2.6)
**Method of transportation to the hospital, n (%)**		
	Ambulance	1 (0.9)
	Car	25 (21.7)
	Electric scooter	1 (0.9)
	Family member or friend	3 (2.6)
	Public transportation	47 (40.9)
	Taxi	4 (3.5)
	Walk	9 (7.8)
	Other	2 (1.7)
	Multiple methods	21 (18.3)
	No answer	2 (1.7)
**Length of time spent checking feet, n (%)**		
	Never	23 (20)
	Less than 1 minute	59 (51.3)
	More than 1 minute	30 (26.1)
	No answer	3 (3)
**Uses a mirror to check the bottom of their feet, n (%)**		
	Yes	16 (13.9)
	No	99 (86)
**Frequency of doctors visits about feet, n (%)**		
	Every week	1 (0.8)
	Every month	5 (4.3)
	A few times a year	61 (53)
	Only when I am sick	32 (27.8)
	Never	13 (11.3)
	No answer	3 (2.6)
**Uses Glucometer, n (%)**		
	Yes	108 (93.9)
	No	7 (6.1)
**Owns Cellphone, n (%)**		
	Yes	94 (81.7)
	No	21 (18.2)
**Would use mHealth to check their feet, n (%)**		
	Yes	86 (74.7)
	No	29 (25.2)

^a^Measured on a scale of 1 to 10, where 10 is considered very important.

Although most of the study respondents (95/115, 82.6%) visit their doctor a few times a year, 17/115 (14.8%) of patients reported that it was difficult to get to the hospital or to see their doctor. Interestingly, 43/115 (37.4%) respondents said that they were retired or unemployed. We found that the occupations of our survey respondents were primarily in “white collar” sectors—46/115 (40.1%) of respondents listed their career as one of “Finance or business,” “Administration or management,” “Education,” “Engineering or technology” or “Healthcare”—and only 4/155 (3.5%) of respondents considered their occupation to be “General labor.” Mobile phone ownership was widespread (93/115, 80.4%) as was glucometer usage (108/115, 94%). Of the patients surveyed, 68/115 (73.1%) would use a device on their phone to help them check their feet (Table 1).

### Clinician Survey

Responses to the clinician survey were mostly from wound care nurses or nurse practitioners (149/202; 73.8%) and chiropodist or podiatrists (20/202, 10.0%) who have been in practice for more than 5 years (Table 2). Most clinicians see their patients regularly—121/202 (60.8%) see their patients weekly or monthly, but 62/202 (31.2%) of clinicians see their patients only when they have a problem (Table 2). This lack of routine care is perceived to be due primarily to patient barriers (41/202, 20.3%), provider barriers (22/202, 10.9%) or a combination of both (115/202, 57.0%), and many clinicians left survey comments identifying patient financial constraints and education about the importance of foot checking as barriers to more frequent care. The concept of mHealth was unfamiliar to the vast majority of wound care clinicians (145/202, 71.8%), and only 7/202 (3.5%) are currently using mHealth in their practices. However, 161/202 (81.7%) respondents thought that more frequent monitoring through mHealth could improve patient outcomes, and 181/202 (91.4%) would consider using an mHealth approach to monitor or supplement patient monitoring between visits (Table 2). The most frequently identified concerns about mHealth were the reliability of patient-generated data (98/202, 49.3%) and the reliability or accuracy of the technology itself (82/202, 41.2%). Several respondents left comments suggesting that their elderly patients would have trouble managing a new mobile phone–based technology, or have trouble affording such a device.

**Table 2:** Summary of responses by clinicians surveyed (N=202).

Demographic		Responses, n (%)
**Clinician role**		
	Nurse or nurse practitioner	149 (73.8)
	Chiropodist or podiatrist	20 (9.9)
	Family physician	5 (2.5)
	Internal medicine	2 (1.0)
	Surgeon (plastic, orthopaedic, vascular)	0 (0)
	Other	26 (12.9)
**Percentage of practice is devoted to diabetic foot ulcers?**		
	Less than 10%	43 (21.3)
	10-50%	111 (55.0)
	Greater than 50%	48 (23.8)
**Years in practice**		
	Less than 1 year	5 (2.5)
	1-5 years	33 (16.3)
	5+ years	164 (81.2)
**Familiarity with mHealth**		
	Using mHealth within practice	7 (3.5)
	Familiar, but not using mHealth	50 (24.8)
	Not familiar with mHealth	145 (71.8)
**Likelihood to use mHealth in practice**		
	Would use mHealth to supplement care/monitor between visits	88 (43.6)
	Would not change current practice, but would use mHealth as a supplement	93 (46)
	Have concerns about using mHealth	17 (8.4)
**Barriers to seeing patients at ideal frequency**		
	Patient barriers	41 (20.3)
	Provider barriers	22 (10.9)
	Combination of barriers	115 (56.9)
	Other	24 (11.9)

## Discussion

Diabetics carry a heavy burden of illness that requires significant healthcare “work,” including diet and lifestyle planning and tracking, medication adherence, doctor’s appointments, and self-monitoring. In a large cohort study recently completed in Alberta, only 14% of respondents reported checking their feet 6 days a week or more, and only 41% and 34% had their feet checked regularly by a clinician for ulcers or sensory loss, respectively [8]. Given that frequent monitoring (and resulting early detection) of foot ulcers is critical to their effective treatment, we are actively seeking ways to increase the frequency of monitoring patients while balancing their quality of life. The present study sought to understand the practices and health “work” done by a group of patients presenting to a plastic surgery wound clinic at SMH in Toronto for foot monitoring and treatment of DFUs, with the goal of developing an effective mHealth strategy for the diabetic lower extremity following the principles of MDM.

While the average lifetime incidence of DFUs among Canadian diabetics is 15% [9], only 11/115 (9.6%) of our study participants reported a prior DFU and only 3/115 (2.6%) had a prior amputation. Our study participants reported seeing their doctor regularly for both their feet and other concerns, and also reported checking their feet regularly, which has been shown to result in lower rates of DFUs and amputation. In fact, our sample population ranks being in control of their own health as very important, which is in itself a predictor of positive outcomes. Our results may also be reflective of our urban location: Al Sayah et al (2015) found that predictors of clinical monitoring included residing in an urban locations, and there are clear regional differences in amputation rates in the United States, with underserviced and rural communities having a +51.3% higher odds of major amputation, +14.9% higher odds of minor amputation, and +41.4% higher odds of inpatient death (*P*<0.05) than their urban diabetic counterparts [10]. We are clearly missing a vulnerable population who are at high risk for DFUs (and amputations) who cannot get to a clinic in person. It is time to start thinking outside the box to reach them.

Diabetics are a technology-oriented patient population, as 108/115 (94%) of our study population reported using a glucometer to monitor their disease. The Canadian population as a whole is becoming increasingly oriented towards mobile phones: 85.6% of the population owned a mobile phone in 2016, up from 62.9% in 2006 [11]. Of the patients who own a mobile phone, 84/115 (73%) responded that they would be interested in using an app on their mobile phone to help them check their feet. Currently available mHealth tools for diabetes management have been shown to significantly improve the frequency of blood glucose monitoring resulting in fewer hyperglycemic events than control groups [6], suggesting that mHealth can positively affect patient-monitoring frequency. Specific tools for the diabetic lower extremity are lacking, although a search of the Apple and Google Play stores returned several apps that are directed at providing education for diabetic foot screening. Unfortunately, although these apps are available, they are grossly underutilized. Despite estimates that 422 million people worldwide have diabetes (and should be checking their feet), the most highly downloaded app from Google Play had only 500-1000 installs. Part of the reason why so few DFU apps exist is that there are limited objective outcome measures available for the diabetic lower extremity. Patients may also fail to appreciate the health implications of a DFU, and neglect checking their feet in favor of checking their blood glucose regularly. Educational campaigns emphasizing the importance of foot health and efforts to develop strong science for the diagnosis of early stage DFUs are necessary components of any future mHealth strategies.

Our patient survey suggests mHealth may be useful from a patient’s perspective, but we were also interested in the perspective of wound care clinicians. The results of our clinician survey suggest that approximately 1 in 3 patients are only being seen by a clinician once they have already developed a problem. Reasons cited by clinicians for this lack of care are not due to systemic barriers like wait times, but are largely a matter of patient education and engagement. Clinicians also cited financial barriers to mobile phone ownership and lack of comfort with technology among their patient populations, which are barriers that must be considered when developing future mHealth strategies. It could be argued, however, that the cost of ulcer prevention versus amputation should be considered from a public policy making perspective, and that the economics of mHealth are attractive in a publicly-funded healthcare system [12]. Baring the barriers stated, most clinicians would be willing to use an mHealth tool as part of their clinical monitoring.

Despite their willingness however, clinicians did express concern about the reliability of using patient-generated data and relying on pictures alone for wound care (eg, no information on parameters such as wound smell). Furthermore, clinical adoption of mHealth would require the development of fee structures for billing, and mechanisms to ensure patients could be called into the clinic quickly if their condition deteriorated. Regulating technology that is used in healthcare would also require substantial oversight at the national level and on a hospital-by-hospital basis, as there are already many companies vying for space in this potentially lucrative market, some of which have a better understanding of wound care than others. Although work remains to be done before mHealth is ready for widespread use in wound care, in our opinion, these challenges are not insurmountable.

To the authors’ knowledge, this is the largest study evaluating the opinion of patients with DFU on mHealth, albeit in a single hospital population. We also surveyed both sides of the patient-care continuum; both patients and healthcare providers are willing to use mHealth for monitoring of the diabetic lower extremity. Weaknesses of our study include the fact that our patient population was drawn from a single plastic surgery wound clinic. It is unknown what proportion of diabetic foot patients ever present to clinics like this, and thus the external validity of the study may only be applicable to a small subset of patients.

In conclusion, we must find ways to increase foot monitoring frequency and effectiveness in diabetic patients. Using unconventional strategies like mHealth may be feasible but should incorporate educational campaigns to motivate patients and clinicians alike, and should move beyond simply taking a picture of a wound and instead build upon evidence-based outcome measures for foot health like tissue oxygenation, perfusion, and free-radical accumulation.

## Appendix

Multimedia Appendix 1 Supplementary figures.

## Abbreviations

DFU: diabetic foot ulcer
MDM: minimally disruptive medicine
SMH: St. Michael’s Hospital
