# Supplementary material for: Assessing the Need for Mobile Health (mHealth) in Monitoring the Diabetic Lower Extremity
Source: JMIR Mhealth Uhealth. 2019 Apr 16;7(4):e11879. doi: 10.2196/11879 (PMC6488952; doi:10.2196/11879)
Supplement: Multimedia Appendix 1 [file mhealth_v7i4e11879_app1.pdf]

Supplementary Information

Patient ID #________

STUDY QUESTIONNAIRE

Demographic information			
Birthdate (month/year)	Gender	Height (ft)	Weight (lbs)
Calculated age:		Calculated BMI:	
What do you do for a living?			
What type of diabetes do you have?<br>Type 1 ☐ Type 2 ☐ Not sure ☐			

Questions about diabetes	Y	N
Do you take insulin?		
Do you use a glucometer?		
Do you smoke?		
Do you wear glasses or contact lenses?		
**Foot checking practices**	**Y**	**N**
Is it comfortable for you to touch your toes?		
How long do you take when you check your feet?<br>☐ Never check ☐ Less than a minute ☐ Longer than a minute		
Do you use a mirror to check the bottom of your feet?		
Have you ever had a diabetic foot wound (DFU)?		
Have you had any toe or leg amputations?		

Questions about mHealthcare	Y	N
How important is it for you to feel in control of your own health? (circle)<br><br>**The doctor is in charge** 1 2 3 4 5 6 7 8 9 10 **I am in charge**		
Do you own a cell phone?<br>(if yes, brand:<br>)		
If you own a cell phone, does it have a built-in camera?		
If you own a cell phone with a camera, how comfortable are you with using it? (circle)<br><br>**Not comfortable** 1 2 3 4 5 6 7 8 9 10 **Completely comfortable**		
If you own a cell phone with a camera, do you use any apps that require the use of a camera?<br>(if yes, what apps?<br>)		
If you own a cell phone, what do you use it for? (select all applicable)<br><br>☐ Work ☐ Entertainment ☐ Communication ☐ News<br><br>☐ Other (please specify) _________________		
Do you have any concerns about the privacy of data on your phone?		
Would you use a device on your phone to help you check your feet?		
How often do you see your doctor?<br><br>☐ Every week ☐ Every month ☐ A few times a year ☐ Only when I'm sick		
How often do you see your doctor about your feet?<br><br>☐ Every week ☐ Every month ☐ A few times a year ☐ Only when I'm sick		
Is it difficult for you to get to the hospital/your doctor?		

How do you get to the hospital?

☐ Walk ☐ Transit ☐ Drive ☐ Other: ________________

Figure S1: Survey administered to patients. Following informed consent, patients in a plastic surgeon's outpatient wound clinic were asked the above questions by a research assistant in August 2017.

Towards Minimally Disruptive Medicine: Assessing the feasibility of Mobile Healthcare (mHealthcare) in Monitoring the Diabetic Lower Extremity

⊕ PAGE TITLE

1. Please tell us what your clinical role is:

- ◯ Nurse or nurse practitioner
- ◯ Chiropodist/Podiatrist
- ◯ Family physician
- ◯ General Internal Medicine
- ◯ Subspecialty Internal Medicine
- ◯ Plastic surgery
- ◯ Orthopaedic surgery
- ◯ Vascular surgery
- ◯ Other

2. What percentage of your practice is devoted to diabetic foot ulcers (DFUs)?

- ◯ less than 10% of my patients are at risk/have a DFU
- ◯ 10%-50% of my patients are at risk/have a DFU

- over 50% of my patients are at risk/have a DFU

3. How many years have you been in practice?

- Less than 1 year
- 1-5 years
- 5+ years

4. How often do you see diabetic patients to help them monitor their foot health?

- Weekly
- Monthly
- Every 6 months
- Yearly
- When they have a problem

5. In your opinion, what is the barrier to care if you are not able to see patients about their foot health as often as you would like?

- Patient barriers (the patient can't get to the hospital easily, can't take time off work, doesn't think routine foot monitoring is important, etc.)
- Provider barriers (lack of clinical resources, lack of time, etc.)
- A combination of the above
- Other (please specify)

6. Are you familiar with the concept of mobile Healthcare (mHealth)?

- Very familiar- I am using mHealth currently in my practice
- I am familiar with mHealth, but have never used it in my practice
- I am not familiar with mHealth

7. Would you consider using an mHealth approach to monitoring your patients' foot health?

- I would use mHealth to monitor my patients between clinic visits
- I would not change my practice, but would look at a patient's mHealth data as a supplemental source of monitoring

- ◯ I have concerns about using mHealth in my practice

8. Which of the following do you think could represent an advantage to using an mHealth approach to foot monitoring? (check all that apply)

- ☐ Patient benefits: less time spent at doctor's appointments
- ☐ Provider benefits: less clinical time spent on routine monitoring
- ☐ System benefits: reduction in wait times, better use of hospital resources
- ☐ Improved health outcomes due to more frequent monitoring
- ☐ I do not believe there is an advantage

9. If you have concerns about adopting an mHealth monitoring routine into your practice, what are they? (check all that apply)

- ☐ Reliability and/or accuracy of the technology/data collection device
- ☐ Reliability of patient-generated data
- ☐ Patient privacy/confidentiality with remote monitoring
- ☐ Changing your practice model
- ☐ No concerns
- ☐ Other (please specify)

NEW QUESTION

or Copy and paste questions

Figure 2: Survey sent to wound care clinicians registered with Wound Care Canada via their listserv in January 2018.
